# Impact of Gestational and Lactational Live Yeast Supplementation to Sows on Litter Performance, Colostrum and Milk Proteome Profiles

**DOI:** 10.1002/vms3.70580

**Published:** 2025-08-22

**Authors:** Yuechi Fu, Venkatesh P. Thirumalaikumar, Theresa M. Casey, Timothy A. Johnson, Jun Xie, Olayiwola Adeola, Kolapo M. Ajuwon

**Affiliations:** ^1^ Department of Animal Sciences Purdue University West Lafayette USA; ^2^ Purdue Proteomics Facility, Bindley Bioscience Center Purdue University West Lafayette USA; ^3^ Department of Botany and Plant Pathology Purdue University West Lafayette USA; ^4^ Department of Statistics Purdue University West Lafayette USA

**Keywords:** colostrum, live yeast, maternal transfer, milk, proteome, piglet

## Abstract

This study investigated the effects of dietary live yeast (LY) supplementation to sows during late gestation and lactation on sow and litter performance indices and colostrum and milk proteome profiles. On Day 77 of gestation, 20 sows were allotted to each of 2 dietary treatments: without (control) or with LY (*Saccharomyces cerevisiae*) supplementation at 0.05% of diet during gestation and 0.1% during lactation. Sow performance was recorded on Days 77 and 112 of gestation and Day 19 of lactation. Litter characteristics were recorded at birth and weaning. Colostrum and milk samples were collected on Days 0, 10 and 18 of lactation for shotgun proteomic analysis. Results showed that there was a higher abundance of immune‐associated proteins such as Ig‐like domain‐containing protein and complement proteins (Complement C8 alpha chain and C1q domain‐containing proteins) in the colostrum and IgG heavy chain in Day 10 milk of LY sows than control sows (*p* < 0.05), whereas the abundance of prostaglandin D synthase was greater in D10 milk of control sows than LY sows (*p* < 0.05). Additionally, milk fat globule EGF and factor V/VIII domain‐containing protein and Niemann–Pick C2 were found to be more abundant in both Days 10 and 18 milk samples from LY sows (*p* < 0.05). Overall, these results showed that dietary gestational and lactational LY supplementation increased the abundance of immune‐associated proteins in colostrum and proteins involved in lipid uptake and processing in mature milk.

## Graphical abstract

Maternal dietary live yeast supplementation during late gestation and lactation increased the abundance of immune‐associated proteins in colostrum and proteins involved in lipid uptake and processing in mature milk.

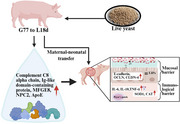

**Abbreviations**
ADFI: average daily feed intake
ADG: average daily gain
ApoE: apolipoprotein E
Ig: immunoglobulin
LY: live yeast
MFGE8: milk fat globule EGF and factor V/VIII domain containing
MS: mass spectrometry
NPC: Niemann–Pick C
PGD: prostaglandin D

## 1 Introduction

Sow milk is a complex and dynamic nutrient source that profoundly affects the early development of the immune system and the growth of piglets before weaning (Kim 2013; Maciag et al. 2022). Maternal nutrition affects milk composition and yield, and thus, nutritional intervention represents an opportunity to improve piglet survival and early growth. Consumption of sufficient amounts of colostrum and milk confers developmental advantages to piglets, and this has been shown to lead to long‐term protection against disease, improved growth performance and increased resilience against various stressors later in life (Declerck et al. 2016). In particular, colostrum provides suckling piglets with passive immunity in the form of maternal immunoglobulins, primarily IgG (Rooke and Bland 2002). Additionally, colostrum and milk contain bioactive components, including functional proteins such as growth factors, antimicrobials and antioxidants, and serve as sources of amino acids to meet the physiological demands of neonatal piglets for the early establishment of resilience and for protection during weaning transition.

In recent years, shotgun proteomics has increasingly been used as a tool to discover the identities and functions of milk proteins due to its robustness and capacity for detecting, identifying and characterizing many functional proteins simultaneously (O'Donnell et al. 2004). With this technique, it has been shown that colostrum and milk contain a plethora of proteins which promote the maturation of the immune system and the gastrointestinal tract of piglets (Bradshaw et al. 2021). It is noteworthy that milk composition and proteomes undergo dynamic changes during the lactation period depending upon the maturity of the mammary gland, and these changes affect the functional properties of milk (Bradshaw et al. 2021). These time‐dependent changes likely reflect the developmental needs of the neonate during the different phases of milk production. Therefore, it is essential to characterize milk proteomes at different stages of lactation to identify proteins that may regulate neonatal growth.

Our previous findings suggested that maternal live yeast (LY) supplementation altered the expression of antioxidant and immune‐regulatory genes in the intestinal mucosa of suckling piglets (Fu et al. 2024). Therefore, we hypothesized that maternal LY supplementation would affect piglet gut health through proteins that are differentially regulated in the colostrum and milk of LY‐supplemented sows compared to control sows. The objective of this study was to evaluate the impact of maternal dietary LY supplementation on the proteome profile of sow colostrum and milk, as well as on sow and piglet performance, with the goal of deepening understanding of the underlying mechanisms by which dietary LY supplementation to sows influences piglet intestinal development.

## 2 Materials and Methods

### 2.1 Animals, Diets and Management

On Day 77 of gestation, a total of 40 crossbred sows (Landrace × Duroc or Landrace × Yorkshire) with similar body weight were randomly assigned to 2 dietary groups (Control, *n* = 20 and LY, *n* = 20) based on their expected farrowing date (average gestation length: 115.5) and parity (average parity: 2.2). The groups received either a basal diet (control) or the basal diet supplemented with a nominal dose of LY at 0.5 g/kg during gestation and 1.0 g/kg during lactation (*Saccharomyces cerevisiae*, 2 × 10^10^ CFU/g, Vistacell, AB Vista, Marlborough, Wiltshire, UK). The actual amount of LY consumed by the sows could not be determined, as the yeast counts in the diets were not analysed. The basal diet (Table 1) was formulated to meet or exceed the nutrient requirements for primiparous sows according to the National Research Council (NRC 2012). The management of the 40 sows from Days 77 to 111 of gestation and during lactation, and cross‐fostering within treatment groups for equalizing litters to 10 or 11 piglets per sow within the first 48 h postpartum, were described in Fu et al. (2024).

**TABLE 1:** Ingredient composition and nutrient levels of gestation and lactation diets (%, as‐fed basis^a^).

Ingredient, %	Gestation	Lactation
Corn	76.95	57.62
SBM, 47.5% CP	17.50	35.00
Swine grease	1.20	3.00
Limestone	1.69	1.63
Monocalcium phosphate	1.30	1.50
Sow vitamin premix^b^	0.15	0.15
Choline chloride (60%)	0.10	0.10
Rovimix‐CarniChrom^c^	0.01	0.01
Phytase^d^	0.10	0.10
NaCl	0.50	0.50
Clarify^e^	0.21	0.10
Defusion Plus^f^	0.25	0.25
Availa Zn^g^	0.04	0.04
Total	100.00	100.00
Calculated composition		
ME, kcal/kg	3286.3	3352.8
CP, g/kg	146.9	214.5
Ca, g/kg	9.0	9.6
Total P, g/kg	5.8	7.2
STTD^h^ P, g/kg	3.7	4.5
SID Lys^i^, g/kg	6.0	10.3

^a^ Live yeast (*Saccharomyces cerevisiae*, 2 × 10^10^ CFU/g, Vista Cell, AB Vista, Marlborough, Wiltshire, UK) was added to the control diets as a replacement for corn in a premix to supply 0.05% and 0.1% for gestation and lactation, respectively.

^b^ Sow vitamin premix, Provimi, Lewisburg, OH. Provided per kg of diet: vitamin A, 11,161 IU; vitamin D_3_, 2545 IU; vitamin E, 66 IU; vitamin K, 1.42 mg; riboflavin, 6.6 mg; pantothenic acid, 23.6 mg; niacin, 44.2 mg; B_12_, 31 µg; biotin, 0.44 mg; folic acid, 1.62 mg; thiamine, 0.25 mg; pyrdoxine‐B_6_, 0.25 mg; iron, 129 mg; zinc, 125 mg; manganese, 60 mg; copper, 20 mg; iodine, 1.26 mg; selenium, 0.3 mg; cobalt, 0.02 mg; calcium, 950 mg; sodium, 800 mg, chloride, 1200 mg; phytase, 371 FTU and estimated 0.12% phosphorus release from the phytase.

^c^ Rovimix‐CarniChrom, DSM Nutritional Products, NJ.

^d^ Phytase (Quantum Blue, AB Vista, Marlborough, UK) premix was added at 1 g/kg to supply 500 FTU/kg phytase.

^e^ ClariFly Larvicide 0.67%, Central Life Sciences, Schaumberg, IL.

^f^ Defusion Plus preservatives, Provimi, Lewisburg, OH.

^g^ Availa Zn 120, Zinpro, Eden Prairie, MN.

^h^ Standardised total tract digestible.

^i^ Standardised ileal digestible lysine.

### 2.2 Sample Collection

Individual body weight, backfat thickness, loin depth, body condition score and rectal temperature were measured for sows on Days 77 and 112 of gestation and the end of lactation (Day 19, average lactation length: 19.1). Briefly, backfat thickness and loin depth were measured at the last rib, approximately 6–7 cm from the midline on both the left and right sides using an ultrasonic scanner (Aloka SSD 500 V, Aloka Co. Ltd., Tokyo, Japan) as described by Li et al. (2021). Rectal temperatures were determined by using a digital thermometer. The body condition score was assessed using a caliper placed at the last rib of each sow (Knauer and Baitinger 2015). The caliper measurements were categorized into a 0–5 scoring system, where 0 = 0–5 mm, 1 = 5–10 mm, 2 = 10–15 mm, 3 = 15–20 mm, 4 = 20–25 mm and 5 = 25–30 mm.

Feed intake (Assured Automation, model WM‐Pd‐050, Roselle, NJ, USA) was recorded weekly during lactation to calculate the average daily feed intake (ADFI). After parturition, the total number of piglets born, the number of born alive and the number of stillborn were recorded for each sow. Piglet weights were recorded individually within 24 h of parturition and at weaning (average weaning age: 19 days), and average daily gain (ADG) was calculated.

A minimum of 30 mL of colostrum and milk samples were collected from sows (*n* = 6) on Days 0 (within 2 h postpartum, one sample was collected at 6 h postpartum due to a technical issue), 10 and 18 of lactation by manually milking functional mammary glands. Prior to milk collection, piglets were removed from sows for 1 h, and 1 mL of oxytocin (VetOne; Boise, ID, USA; 20 USP/mL) was injected into the vulva of the sows to stimulate milk letdown. After collection, milk was mixed to homogeneity and aliquoted into 2 mL conical tubes and stored at −80°C until further analysis.

### 2.3 Analysis of IgG and IgA in Colostrum and Milk

The concentrations of immunoglobulin (Ig)G and IgA were measured using the respective commercial ELISA kits IGG‐9 and IGA‐9 (Life Diagnostics Inc., West Chester, PA, USA) following the manufacturer's protocols.

### 2.4 Protein Extraction and In‐Solution Trypsinization

**FIGURE 1:**
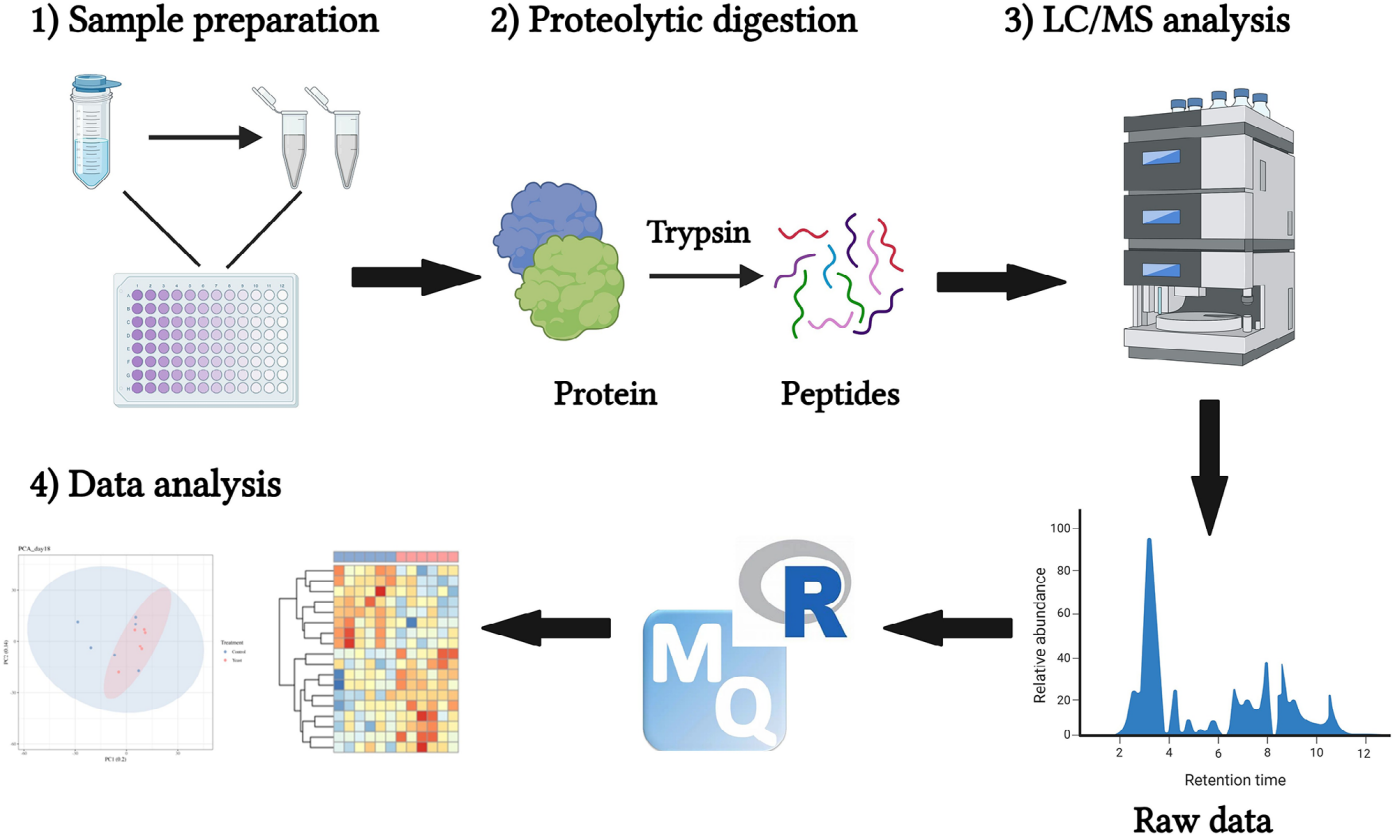
Workflow of the proteomic analysis. LC/MS, liquid chromatography mass spectrometry. *Source*: Created by BioRender.com.

The workflow for the proteomic analysis is shown in Figure 1. Protein concentrations of the colostrum and milk samples (*n* = 6 at each time point) were determined using a Pierce BCA protein assay (Catalog #: 23227, Thermo Scientific, Waltham, MA, USA). A total of 25 µg of protein from each sample was taken for in‐solution digestion. The protein was precipitated using 4 volumes of ice‐cold acetone (kept at −20°C) and digested as described previously (Setyabrata et al. 2023). Briefly, samples were reduced using dithiothreitol and alkylated using iodoacetamide, followed by the addition of sequence grade Lyc‐C/Trypsin (Promega Corporation, Madison, WI, USA) to cleave the proteins into peptides. All digestion was carried out in a Barocycler NEP2320 (Pressure BioSciences Inc., Easton, MA, USA) following the manufacturer's instructions. The samples were then desalted over C18 MicroSpin columns (The Nest Group Inc., Ipswich, MA, USA), dried and re‐suspended in a resuspension solution of 5% acetonitrile and 0.1% formic acid.

### 2.5 Liquid Chromatography–Tandem Mass Spectrometry (MS) Analysis

One microgram peptide from each sample was quantified and loaded onto the column. Peptides were analysed using a Vanquish Neo UHPLC System (Thermo Scientific, Odense, Denmark) coupled to an Orbitrap Exploris 480 mass spectrometer (Thermo Scientific, Waltham, MA, USA), as described previously (DeMarco et al. 2024). Briefly, reverse‐phase peptide separation was performed using a two‐stage column setup, consisting of a trap column (300 µm × 5 mm C18 PepMap 100) and a 75 µm × 50 cm reverse‐phase IonOpticks analytical column (Thermo Scientific, Waltham, MA, USA), heated to 50°C. Peptides were separated by the analytical column at a flow rate of 400 nL/min using a 130‐min gradient. The linear gradient began at 2% solvent B (80% acetonitrile and 0.01% formic acid in MS‐grade water) and reached 25% solvent B in 80 min, 35% solvent B in 100 min, 45% solvent B in 105 min and 95% solvent B in 120 min. The column was held at 95% solvent B for 5 min before reverting to 2% solvent B, where it was equilibrated to regain the starting conditions. Samples were then injected into the Orbitrap Exploris 480 through the Nanospray Flex Ion Source (Thermo Scientific, Waltham, MA, USA). The MS scan range was 350–1650 *m*/*z* at a resolution of 60,000. The automatic gain control target was set to standard, with the maximum injection time kept at auto and a dynamic exclusion of 30 s. The ddMS2 data were acquired using the Orbitrap mass analyser at a resolution of 15,000, with an isolation window *m*/*z* 1.6. Data were collected in the centroid mode.

### 2.6 Proteomics Data Analysis

Liquid chromatography tandem MS raw data were analysed using MaxQuant software (version 2.0.3.0) using the *Sus scrofa* (version 11.1) database downloaded from UniProt (https://www.uniprot.org) with the standard settings. The following parameters were used for the analysis: precursor mass tolerance of 10 ppm; enzyme specificity of trypsin/Lys‐C enzyme allowing up to two missed cleavages; oxidation of methionine as a variable modification and iodoethanol as a fixed modification. False discovery rate of peptide spectral match and protein identification was set to 0.01 (Setyabrata et al. 2023). Proteins were quantified using unique plus razor peptides. To identify proteins that are common and unique between treatments at each time point, Venn diagrams were generated using proteins present in at least 50% of the samples across treatment groups at each time point. Missing values in samples were imputed using Perseus (version 2.0.3.0) with a width of 0.3 and a downshift of 1.8 (Raggi et al. 2023). Label‐free quantification intensity values were then log2 transformed, and differentially abundant proteins between treatments were identified at *p* < 0.05 and visualized using heatmaps in RStudio (version 4.3.1). Quality reports (Figures S1–S3) were generated from Mass Dynamics (https://massdynamics.com/) and RStudio (version 4.3.1).

### 2.7 Statistical Analysis

Data were analysed using Welch's *t*‐test in RStudio (version 4.3.1) for differences between control and LY with sow as the experimental unit. The Shapiro–Wilk test was used to analyse the normality of the data. Data were presented as means ± SEM. Significance was set at *p* ≤ 0.05, and a tendency was defined at 0.05 < *p* ≤ 0.10. Results were illustrated using GraphPad Prism (version 7.03; GraphPad Software Inc., San Diego, CA, USA).

## 3 RESULTS

### 3.1 Effects of Maternal Dietary LY Supplementation on Sow and Litter Performance

Sow performance responses are shown in Table 2. Feeding LY to sows during late gestation and lactation did not affect body weight, backfat thickness, loin depth and body condition score (*p* > 0.05). However, LY sows tended to have a higher rectal temperature on Day 19 of the lactation (*p *= 0.10). Additionally, there were no differences in ADFI during the lactation period.

**TABLE 2:** Effect of maternal dietary live yeast supplementation on sow performance indices.

Items	Groups^a^			
	Control	LY	SEM	*p* value
Body weight, kg				
Day 77 of gestation	213.9	210.3	13.67	0.83
Day 112 of gestation	232.4	226.0	7.68	0.68
Day 19 of lactation	212.7	211.9	13.34	0.96
Backfat thickness, mm				
Day 77 of gestation	20.5	21.9	1.68	0.57
Day 112 of gestation	22.6	23.0	1.67	0.76
Day 19 of lactation	18.4	17.8	0.58	0.46
Loin depth^b^, mm				
Day 77 of gestation	49.4	46.2	2.10	0.23
Day 112 of gestation	46.1	44.5	2.79	0.64
Day 19 of lactation	44.0	44.2	1.34	0.91
Body condition score^c^				
Day 77 of gestation	3.0	3.0	0.20	0.85
Day 112 of gestation	3.1	3.1	0.16	1.00
Day 19 of lactation	2.5	2.6	0.80	0.73
Rectal temperature, °C				
Day 77 of gestation	38.2	38.3	0.40	0.49
Day 112 of gestation	38.0	37.9	0.27	0.73
Day 19 of lactation	38.4	38.8	0.38	0.10
Lactation feed intake, kg				
ADFI	5.87	5.96	0.25	0.81

Abbreviations: ADFI, average daily feed intake; LY, live yeast.

^a^ *n* = 20 sows for Control and LY treatments, respectively.

^b^ Loin depth = Total loin depth–backfat thickness.

^c^ Caliper measurements were categorized into a 0–5 scoring system: 0 = 0–5 mm, 1 = 5–10 mm, 2 = 10–15 mm, 3 = 15–20 mm, 4 = 20–25 mm, 5 = 25–30 mm.

No treatment effects were found on litter performance traits, including the total number of piglets born and the number of born alive, stillborn and litter size at weaning (Table 3). Piglet performance was also similar between the treatment groups, including individual body weight at birth and weaning, as well as ADG (Table 3).

**TABLE 3:** Effect of maternal dietary live yeast supplementation on litter performance.

	Groups^a^			
	Control	LY	SEM	*p* value
Number of piglets/L				
Total born^b^	12.8	12.4	0.92	0.84
Born alive	11.7	10.8	0.61	0.54
Stillborn	1.1	1.6	0.37	0.42
After cross‐fostering	10.7	10.5	0.16	0.60
At weaning^c^	9.3	8.9	0.29	0.26
Piglet performance				
Average body weight at birth^d^, kg	1.78	1.69	0.09	0.47
Average body weight at weaning, kg	6.76	6.26	0.24	0.14
Average daily gain^e^, kg	0.26	0.25	0.01	0.67

Abbreviation: LY, live yeast.

^a^ *n* = 20 sows for control and LY treatments, respectively.

^b^ Total born was calculated as the sum of the number born alive and the number of stillborn.

^c^ A total of 52 piglets, selected from 20 sows (10 per treatment), were slaughtered during lactation. Eight piglets were preplanned for slaughter and counted in the litter size at weaning.

^d^ Average body weight at birth was collected during Day 1 processing.

^e^ Pigs were weaned at an average age of 19 days.

### 3.2 Effects of Maternal Dietary LY Supplementation on Colostrum and Milk Proteomes

**FIGURE 2:**
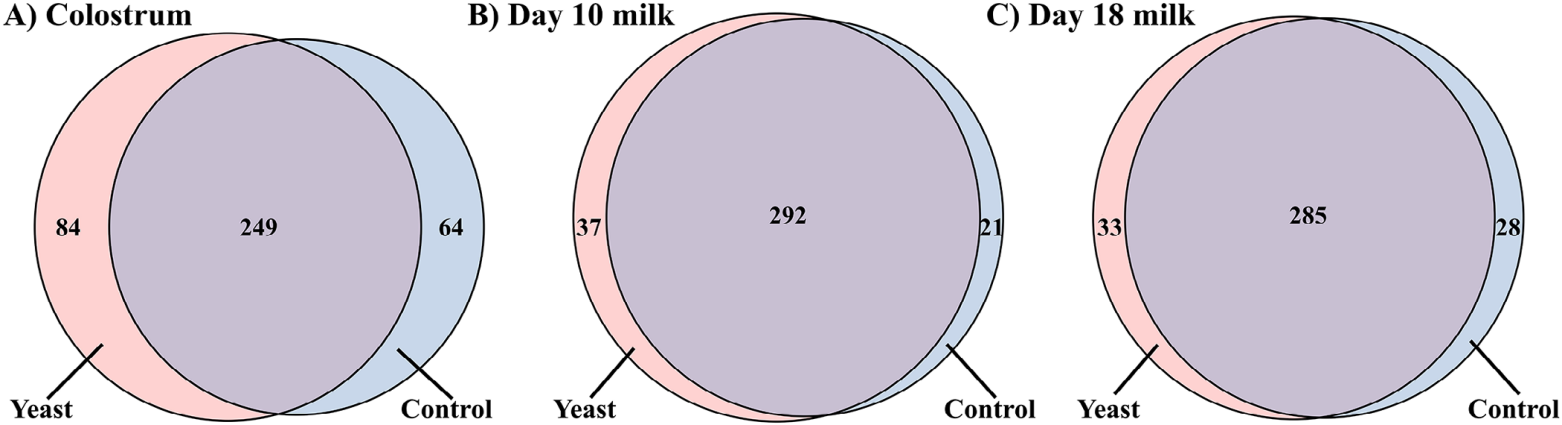
Venn diagram of the distribution of proteins (detected in at least 50% of samples) that are common and unique in (A) colostrum, (B) Day 10 milk and (C) Day 18 milk between control (blue) and yeast (red) sows.

**FIGURE 3:**
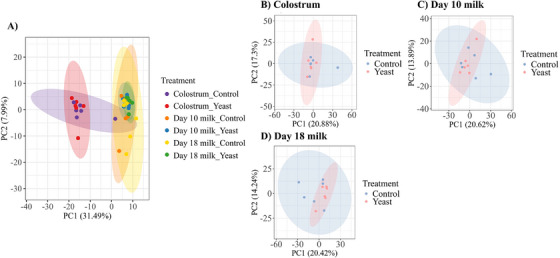
Principal component analysis of (A) overall, (B) colostrum, (C) Day 10 milk and (D) Day 18 milk in control (blue) and yeast (red) sows.

**FIGURE 4:**
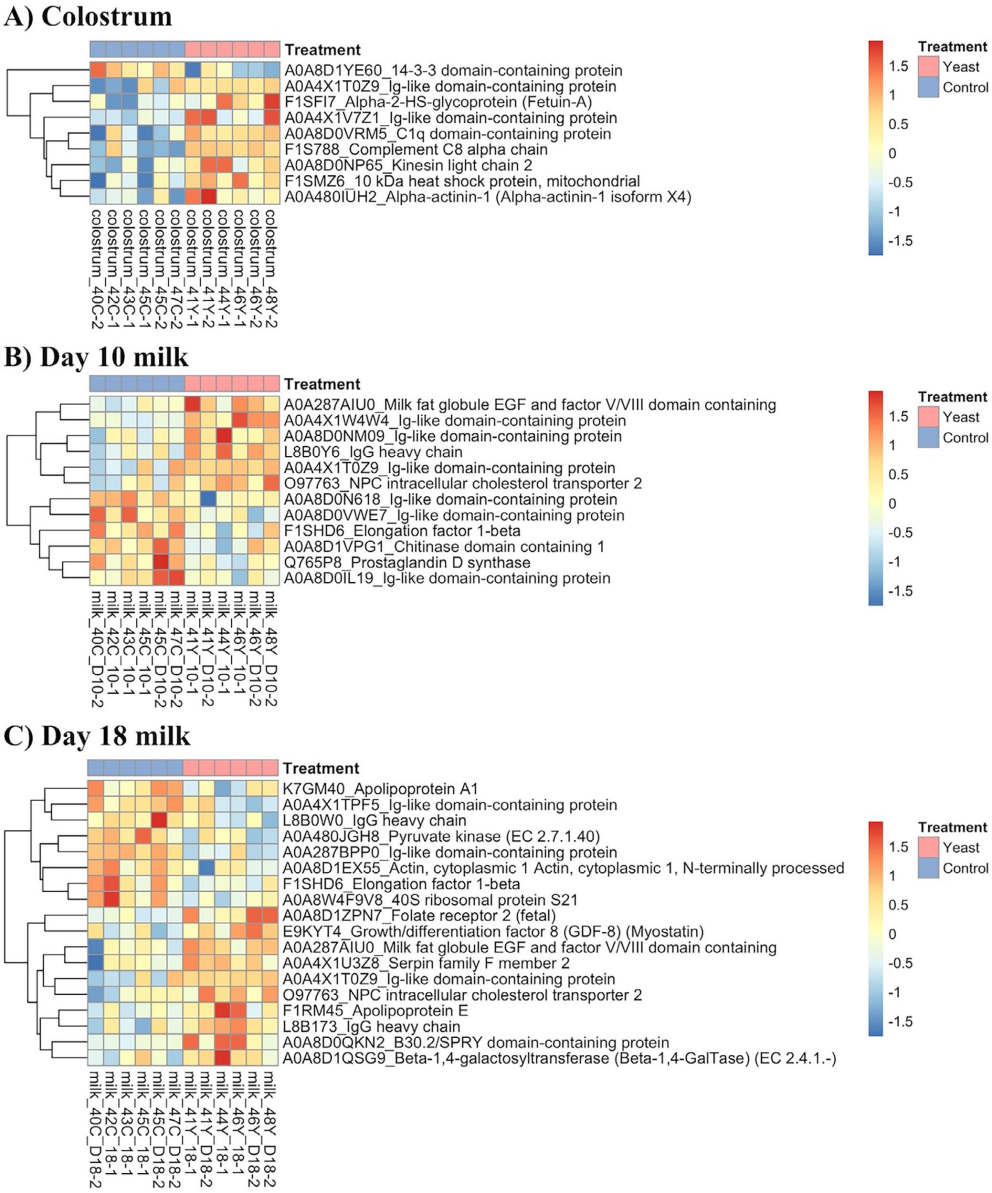
Heatmaps illustrate differentially abundant proteins in (A) colostrum, (B) Day 10 milk and (C) Day 18 milk between control (blue) and yeast (red) sows (*n* = 6). The gradient bar represents *z*‐score values.

A total of 397 proteins were detected in at least 50% of the samples in the treatment groups, of which 249, 292 and 285 proteins were commonly expressed across all groups in colostrum, Days 10 and 18 milk, respectively (Figure 2). Principal component analysis revealed that the day of lactation affected milk proteome profiles (colostrum samples were clustered more distantly from Days 10 to 18 milk samples, but no clear difference was observed between control and LY sows (Figure 3). There were 9, 12 and 18 differentially abundant proteins in the colostrum, Days 10 and 18 milk, respectively, between control and LY sows (Figure 4, *p* < 0.05). Proteins that were more abundant in the colostrum of LY sows were associated with immunity, including Ig‐like domain‐containing protein and complement proteins (Complement C8 alpha chain and C1q domain‐containing proteins, Figure 4A). Similarly, proteins found to be more abundant in Day 10 milk of LY sows included IgG heavy chain, milk fat globule EGF and factor V/VIII domain containing (MFGE8) and Niemann–Pick C2 (NPC2), whereas prostaglandin D (PGD) synthetase and elongation factor 1β were more abundant in Day 10 milk of control sows (Figure 4B). Additionally, proteins that included growth/differentiation factor‐8, MFGE8, serpin family F member 2, NPC2, apolipoprotein E, IgG heavy chain and beta‐1,4‐galactosyltransferase were found to be more abundant in Day 18 milk of LY sows, whereas pyruvate kinase and elongation factor 1β were more abundant in Day 18 milk of control sows compared to LY sows (Figure 4C).

### 3.3 Effects of Maternal Dietary LY Supplementation on IgG and IgA Levels in Colostrum and Milk

**FIGURE 5:**
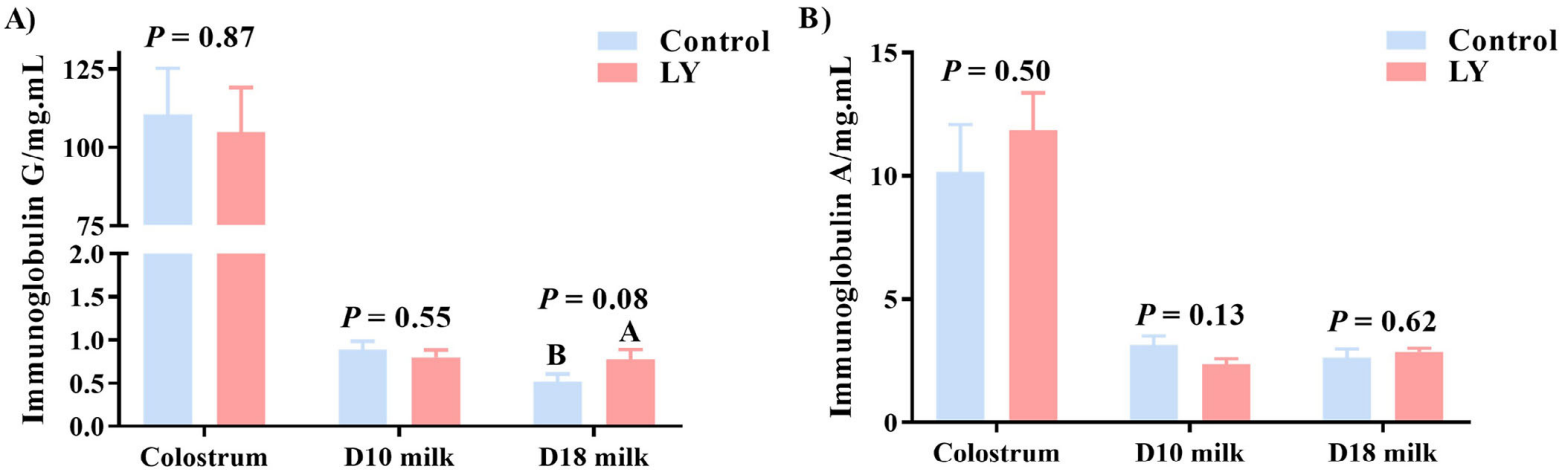
Effect of maternal dietary live yeast supplementation on the concentrations of IgG and IgA in colostrum, Day 10 milk and Day 18 milk (*n* = 6). D, day; Ig, immunoglobulin; LY, live yeast. ^A,B^ Indicates trend differences (0.05 < P ≤ 0.10).

Sows that received diets supplemented with LY tended to have a higher concentration of IgG in Day 18 milk (Figure 5, *p* = 0.08), but no differences were observed in the concentrations of IgG and IgA in colostrum and Day 10 milk between the treatment groups (Figure 5).

## 4 Discussion

Research evidence has shown that supplementing sows with LY and yeast‐based products during gestation and lactation benefits piglet health and development (Broadway et al. 2015). Our earlier findings demonstrated that maternal LY supplementation enhanced intestinal development in suckling piglets (Fu et al. 2024) and improved growth performance and nutrient digestibility in nursery pigs (Lu et al. 2019). Immediately after birth, piglets begin consuming colostrum and milk, which serve as sources of various immunomodulatory factors, including immunoglobulins, cytokines and growth factors essential for stimulation of the development of the immune system and gastrointestinal tract (Dzidic et al. 2018). Thus, elucidating the mechanisms by which maternal LY supplementation exerts these beneficial effects, through accurately determining its impact on the abundance of functional proteins in milk and colostrum, is essential for understanding how LY may be used to enhance piglet growth and development.

In the current trial, maternal LY supplementation had no effect on sow body weight, backfat thickness, loin depth or body condition score on Days 77 and 112 of gestation and Day 19 of lactation, nor did it affect feed intake during lactation. These results align with Xia et al. (2022) and Le Flocʹh et al. (2022), who similarly found no effects of LY supplementation on sow body weight, backfat thickness or feed intake when administered from early or mid to late gestation through lactation. Additionally, LY had no impact on litter performance traits, including total born, born alive, stillborn and litter size. Given that litter size is primarily determined by fertilization rate and early embryonic survival (Bolet 1986), dietary changes in late pregnancy are unlikely to influence this parameter. Similarly, no differences in piglet ADG during suckling or body weight at weaning were observed, suggesting that although LY may enhance intestinal development (Fu et al. 2024), these benefits may not be immediately translated into measurable improvements in piglet growth performance during this early growth stage. However, any effects could potentially become more apparent in later stages of life such as the nursery or grower finisher stages. Nevertheless, conflicting results have been reported on the effect of LY on the litter characteristics of sows. Peng et al. (2020) reported that sows fed an LY‐containing diet had fewer number of stillborn and low body weight piglets. Similarly, maternal yeast nucleotide supplementation was found to increase litter weight and ADG (Gao et al. 2021). The discrepancies between these results and the present findings may be attributable to multiple factors, including type of yeast strain used, the duration and level of yeast inclusion, sample size, genetics and parity of sows. Additional studies are needed to clarify the impact of these factors on sows supplemented with LY.

Maternal nutrition influences both the qualitative and quantitative aspects of colostrum and milk, including abundance of functional proteins and nutrient composition (Kim 2013). Current results suggested that the colostrum and milk of LY sows exhibited variations in protein abundance at different stages of lactation compared to control sows. Specifically, there were 9, 12 and 18 differentially abundant proteins in colostrum, Days 10 and 18 milk samples, respectively, in response to maternal dietary LY supplementation. Notably, compared to control sows, eight proteins were more abundant in the colostrum of LY‐fed sows, including three immune‐associated proteins: Ig‐like domain‐containing protein, C1q domain‐containing protein and Complement C8 alpha chain. The enrichment of these immune‐associated proteins underscores the vital role of colostrum as a source of passive immunity and neonate immune development, providing piglets with immunoglobulins, immune cells and cytokines that are essential for neonatal immune system development and early defence against pathogens (Quesnel 2011; Poonsuk and Zimmerman 2018). As the epitheliochorial porcine placenta does not permit the transfer of immunoglobulins from maternal to foetal circulation, the intake of IgG‐rich colostrum in the first 24 h after birth establishes passive immunity in piglets, as these immunoglobulins are absorbed through the small intestine and enter systemic circulation prior to gut closure (Laber et al. 2002). Thus, the elevated expression of immune‐related proteins in the colostrum of LY sows supports their role in the acquisition of passive immunity and the development of immunocompetence in piglets during the suckling phase (Yang et al. 2015). Although direct ELISA measurements did not reveal differences in IgG concentrations, this may be attributed to the single time point of sample collection, as immunoglobulin levels, particularly IgG and IgA, can fluctuate considerably within the first 12–24 h postpartum (Szabó et al. 2025). Taken together, these findings suggest that maternal LY supplementation modulates the immunological properties of colostrum and milk, potentially supporting the developmental needs of piglets more effectively than the colostrum and milk of control sows. Such proteomic alterations are likely mediated through the gut‐immune‐mammary axis, influenced by the immunomodulatory effects of yeast cell wall components, particularly β‐glucans and mannooligosaccharides, which may affect maternal immune responses, microbial composition and mammary gland secretory function (Li et al. 2022).

Furthermore, milk fat globule EGF and factor V/VIII domain‐containing protein (MFGE8) and Niemann–Pick C2 (NPC2) were more abundant in both Days 0 and 18 milk samples from LY sows. As a primary protein in the milk fat globule membrane, MFGE8 (aka lactadherin) functions in the transport of essential lipids to neonates. It also has immunomodulatory effects, including the clearance of apoptotic cells and acting as an anti‐inflammatory protein, both of which are crucial for intestinal health (Yi 2016). Studies have shown that the administration of recombinant MFGE8 ameliorated colitis by reducing intestinal inflammation in mice (Aziz et al. 2009). Additionally, our earlier findings suggested that maternal LY supplementation enhanced the expression of tight junction proteins and primed the immune system (increased mRNA expression of both pro‐ and anti‐inflammatory cytokines) in the small intestines of piglets on postnatal Days 10 and 18 (Fu et al. 2024). Thus, elevated levels of MFGE8 in Days 10 and 18 milk samples from LY sows may confer immunological advantages to piglets by promoting intestinal health and reducing inflammatory challenges during early life. This effect is particularly beneficial during the transition from colostrum to mature milk, as the nutritional and immunological requirements of piglets increase during mid and late lactation (Huting et al. 2021). Additionally, the higher abundance of NPC2 in milk of LY sows may enhance fat uptake and metabolism in suckling piglets due to its central role in the intracellular processing of cholesterol (Storch and Xu 2009). This is important postnatally, as lipids account for 50% of the calories in milk and serve as an important nutrient source during this stage. In contrast, during the in utero period, glucose is the primary calorie source for the developing foetus (Girard et al. 1992). In Day 10 milk of control sows, PGD synthase, an enzyme crucial for converting prostaglandin H to PGD, was more abundant than that of LY sows. Elevated levels of PGD can reflect oxidative stress and inflammation, which are characteristic of mastitis in dairy cows (Baeker et al. 2002). The enrichment of PGD synthase in milk from control sows compared to LY may suggest that LY exerts an anti‐inflammatory effect, potentially reducing the need for PGD production. LY has been shown to modulate immune function by increasing beneficial microbiota and enhancing host defence mechanisms (Broadway et al. 2015). Consequently, the lower PGD synthase could reflect a more balanced immune environment (less prostaglandin‐mediated inflammation) in Day 10 milk of LY sows.

Additionally, compared to colostrum and Day 10 milk, the increased number of differentially abundant proteins between control and LY sows in Day 18 milk may be attributable to the prolonged duration of LY supplementation and the different physiological demands across lactation stages. Proteomic analysis showed that Day 18 milk of LY sows had a greater abundance of IgG heavy chain, consistent with direct measurements from ELISA showing a trend toward elevated IgG levels, which may enhance immune support for piglets during late lactation. Furthermore, similar to the observations in Day 10 milk of LY sows, there was a higher abundance of proteins involved in lipid uptake and processing, including MFGE8, NPC2 and apolipoprotein E (ApoE) in Day 18 milk of LY sows. Apolipoprotein E is essential for lipid metabolism and transport, particularly the transport of triglycerides, cholesterol and fat‐soluble vitamins, facilitating lipid digestion and absorption in neonates, thereby supporting their growth and development (Vatassery et al. 2006; Mahley 2016). The enrichment of these proteins may indicate improved lipid uptake in piglets from LY‐supplemented sows, which may be vital for meeting their energy demands.

In summary, maternal LY supplementation induced stage‐specific alterations in the abundance of functional milk proteins, with an increase in the abundance of immune‐related proteins in colostrum during early lactation and a higher abundance of proteins associated with lipid uptake and processing in mature milk during late lactation. Consequently, these changes in colostrum and milk proteomes may enhance neonatal immune competence, metabolic function and intestinal maturation. This study adds to the growing evidence of the critical role of maternal nutritional intervention in shaping colostrum and milk quality and developmental programming of offspring.

## 5 Conclusion

The current results indicate that although dietary LY supplementation to sows during late pregnancy and lactation has minimal impacts on sow and litter performance, it may increase the abundance of functional proteins associated with immunity, lipid uptake and processing that may lead to enhancement of intestinal development in suckling piglets. Therefore, this study advances our understanding of the potential mechanisms through which maternal LY supplementation may confer health and developmental benefits to piglets.

## Author Contributions

**Yuechi Fu**: conceptualization, data curation, formal analysis, methodology, writing – original draft, writing – review and editing. **Venkatesh P. Thirumalaikumar**: data curation, writing – review and editing. **Theresa M. Casey**: software, supervision, writing – review and editing. **Timothy A. Johnson**: writing – review and editing. **Jun Xie**: supervision, writing – review and editing. **Olayiwola Adeola**: supervision, writing – review and editing. **Kolapo M. Ajuwon**: conceptualization, funding acquisition, project administration, supervision, writing – review and editing. All authors have read and agreed to the published version of the manuscript.

## Ethics Statement

All procedures followed the ethical principles and were approved by Purdue University Institutional Animal Care and Use Committee (number: 1111000145, West Lafayette, IN, USA).

## Conflicts of Interest

The authors declare no conflicts of interest.

## Peer Review

The peer review history for this article is available at https://www.webofscience.com/api/gateway/wos/peer‐review/10.1002/vms3.70580.

## Supporting information



Supporting File: vms370580‐sup‐0001‐Figures S1‐S3.docx

## Data Availability

Data are available from the corresponding author upon request.
