# Supplementary material for: Impact of Gestational and Lactational Live Yeast Supplementation to Sows on Litter Performance, Colostrum and Milk Proteome Profiles
Source: Vet Med Sci. 2025 Aug 22;11(5):e70580. doi: 10.1002/vms3.70580 (PMC12372606; doi:10.1002/vms3.70580)

Figure S1. Quality Report: percentage of proteins detected

**
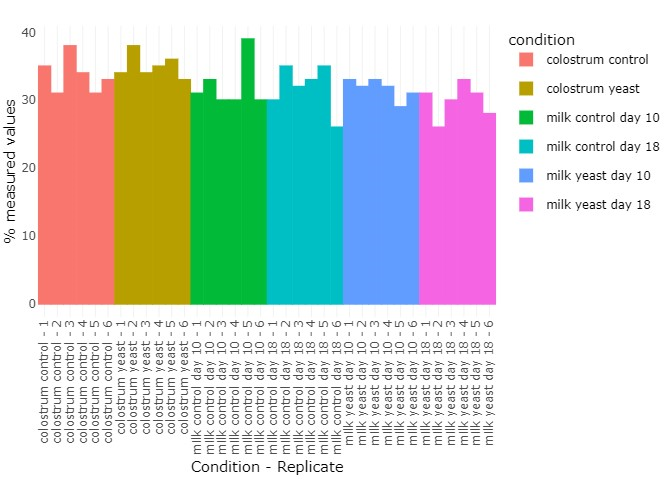
**

Figure S2. Quality Report: Digestion efficiency and peptide spectral matches (PSMs).


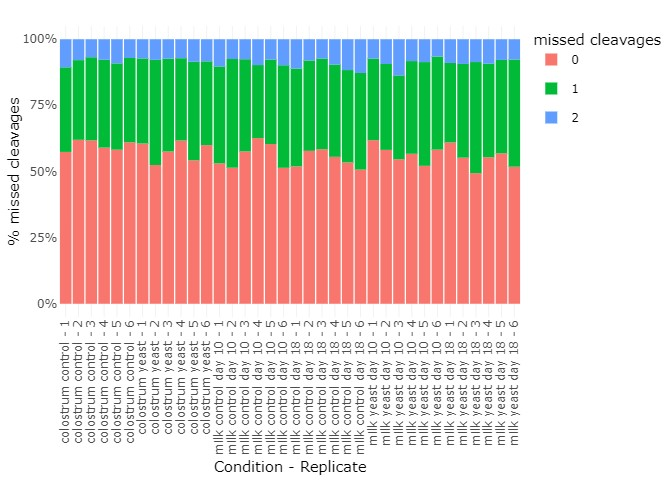


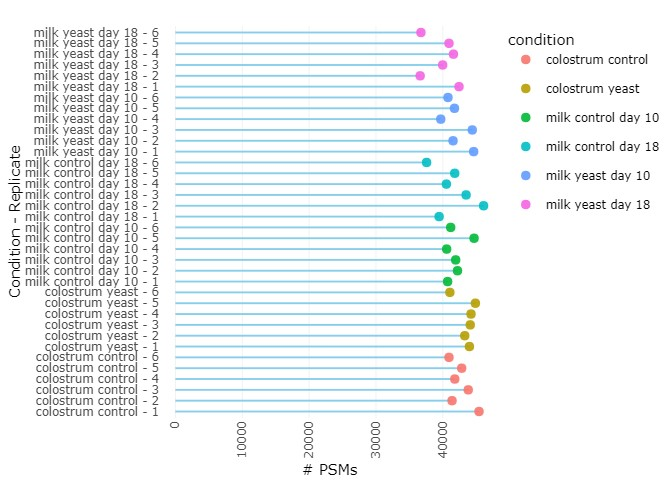


Figure S3. Correlation analysis of Label-free quantification intensity log2 values between treatment and across days.


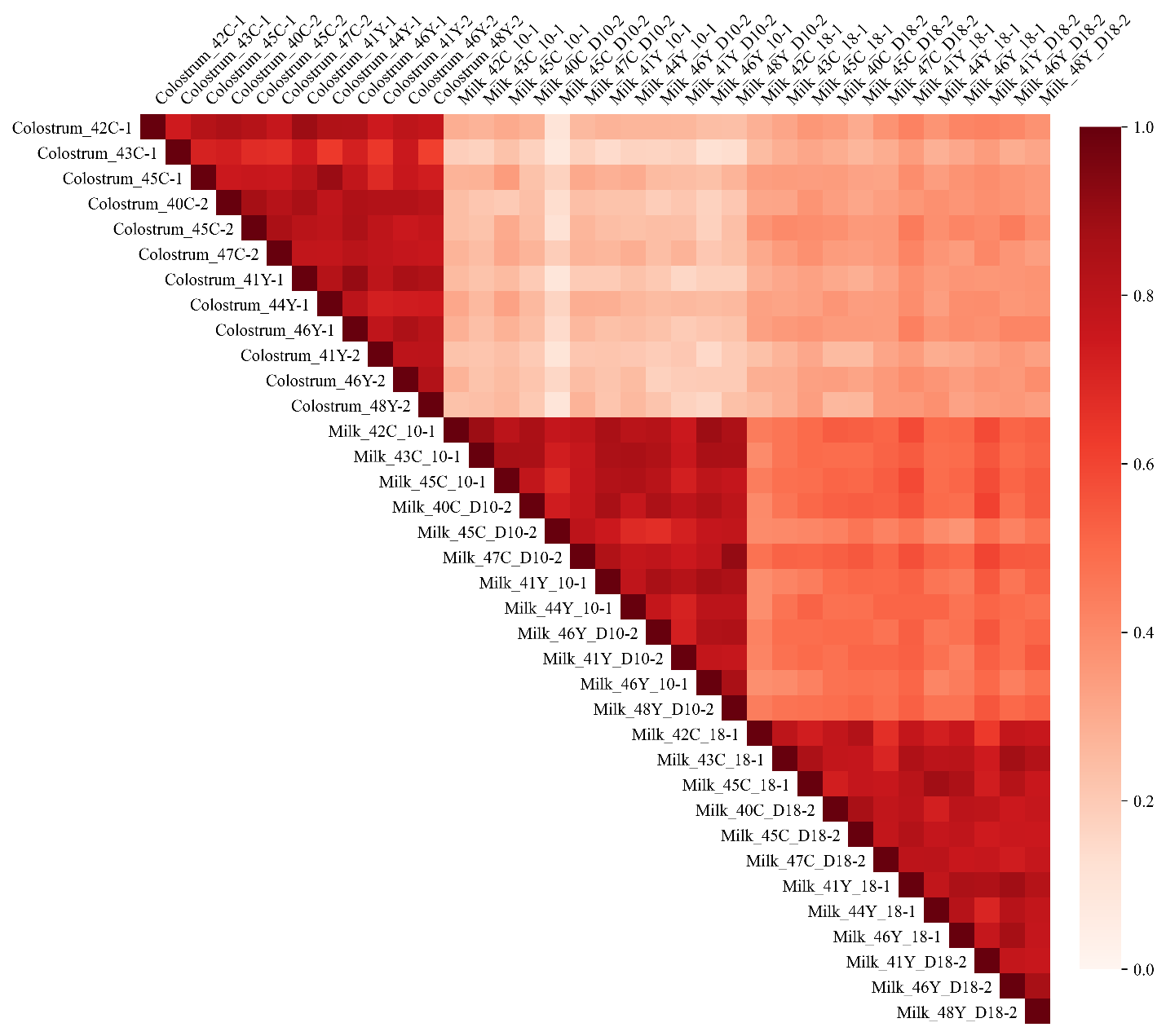

Supplement: Supplementary file 1 — Supporting File: vms370580‐sup‐0001‐Figures S1‐S3.docx [file VMS3-11-e70580-s001.docx]
